# Machine Learning Classification Identifies Cerebellar Contributions to Early and Moderate Cognitive Decline in Alzheimer’s Disease

**DOI:** 10.3389/fnagi.2020.524024

**Published:** 2020-11-03

**Authors:** Muriel M. K. Bruchhage, Stephen Correia, Paul Malloy, Stephen Salloway, Sean Deoni

**Affiliations:** ^1^Advanced Baby Imaging Lab, Hasbro Children’s Hospital, Rhode Island Hospital, Providence, RI, United States; ^2^Department of Pediatrics, Warren Alpert Medical School at Brown University, Providence, RI, United States; ^3^Butler Hospital Memory and Aging Program, Providence, RI, United States; ^4^Department of Human Behavior and Psychiatry, Warren Alpert Medical School at Brown University, Providence, RI, United States; ^5^Department of Neurology, Warren Alpert Medical School at Brown University, Providence, RI, United States; ^6^Maternal, Newborn and Child Health Discovery & Tools, Bill & Melinda Gates Foundation, Seattle, WA, United States

**Keywords:** Alzheimer’s disease, dementia, cerebellum, machine learning, MCI (mild cognitive impairment), mild moderate AD, gray matter (GM), white matter (WM)

## Abstract

Alzheimer’s disease (AD) is one of the most common forms of dementia, marked by progressively degrading cognitive function. Although cerebellar changes occur throughout AD progression, its involvement and predictive contribution in its earliest stages, as well as gray or white matter components involved, remains unclear. We used MRI machine learning-based classification to assess the contribution of two tissue components [volume fraction myelin (VFM), and gray matter (GM) volume] within the whole brain, the neocortex, the whole cerebellum as well as its anterior and posterior parts and their predictive contribution to the first two stages of AD and typically aging controls. While classification accuracy increased with AD stages, VFM was the best predictor for all early stages of dementia when compared with typically aging controls. However, we document overall higher cerebellar prediction accuracy when compared to the whole brain with distinct structural signatures of higher anterior cerebellar contribution to mild cognitive impairment (MCI) and higher posterior cerebellar contribution to mild/moderate stages of AD for each tissue property. Based on these different cerebellar profiles and their unique contribution to early disease stages, we propose a refined model of cerebellar contribution to early AD development.

## Introduction

Alzheimer’s disease (AD) is the most prevalent form of dementia in the developed world (Reitz et al., 2011) and is defined by a progressive decline of a variety of cognitive functions and motor abilities. Accumulating evidence suggests that AD has a lengthy preclinical phase, where brain pathology accumulates and patient function declines, but symptoms are insufficient to warrant a clinical diagnosis of dementia. AD neurodegeneration follows specific topographic patterns of gray and white matter atrophy that emerge during its early stages (Serra et al., 2010). Although the pathobiological basis of these alterations remains unclear, there is increasing evidence that white matter and myelin alterations occur at the earliest stages of the disease and are associated with cognitive decline, potentially preceding gray matter (GM) volume changes and loss (Braak et al., 2000). This has been supported by emerging preclinical (Desai et al., 2010), histological (Zhan et al., 2014) and longitudinal studies documenting white matter changes to be of particular importance for AD stages (Frings et al., 2014), in turn stressing the need to better characterize white matter and myelin change in early stages of cognitive decline in AD.

While the traditionally focus has been placed on neocortical and hippocampal atrophies, recent evidence suggests that the cerebellum undergoes focal atrophy in concert with interconnected cerebral nodes in both AD and fronto-temporal dementia (Guo et al., 2016). Histopathology studies of AD have shown cerebellar amyloid-β oligomers to be affected in a counter-clockwise pattern during disease progression, starting in the posterior cerebellar lobe, and paralleling cerebrum atrophy staging and associated symptom progression (Jacobs et al., 2018).

However, the functional decline is often present before an official AD diagnosis has been reached. For example, 60% of elderly patients with a cognitive decline also suffer from falls; twice more than those without impairment (Davis et al., 2011). In a functional MRI study, falling significantly more often in the elderly has implicated the right cerebellum as a potential region of interest in early cognitive decline and aging (Liu-Ambrose et al., 2008). While the cerebellum has long been associated with motor function, it is also involved in many non-motor functions, including working memory and executive functioning (Schmahmann et al., 2001; Bellebaum and Daum, 2007). Nevertheless, the role of cerebellar white and GM in disease progression and classifying stages of early cognitive decline from typical aging remain a challenge. To address these questions, we used machine learning-based classification on GM anatomical and volume fraction myelin images to investigate their contributions to predict the first two early stages of dementia [mild cognitive impairment (MCI) and mild and moderate AD] when compared with typically aging controls. We further calculate their predictive accuracy within the whole brain and neocortex and compare them with the anterior, posterior, and whole cerebellum to investigate the role of cerebellar white and GM in early AD progression.

## Materials and Methods

### Participants

Forty-three age- and gender-matched participants (15 healthy controls; 17 MCI; and 11 Mild/Moderate AD) were included. Subject group demographics are shown in Table 1. A clinical interview, an assessment of cognitive decline using the Mini-Mental State Examination (MMSE), and clinical dementia rating (CDR) scores were used to assign the participants to the healthy (CDR = 0), MCI (CDR ≤ 0.5), and Mild/Moderate AD (0.5 ≤ CDR ≤ 1.0) groups. The CDR is a reliable and valid measure to assess AD stages (Morris, 1993; Nyunt et al., 2013) and its scores have been shown to usefully predict functional decline and incident dementia (Woolf et al., 2016). The MMSE is a 30-point questionnaire that is routinely used to assess cognitive impairment in clinical and research settings (Pangman et al., 2000). MANOVAs revealed no significant group differences in mean age or gender ratio between groups (*p* > 0.10, Table 1). Genetic screening was performed on individuals with AD and APOE (Apolipoprotein E) status was determined.

**Table 1:** Participant demographics.

	Healthy aging	Mild cognitive impairment	Mild to moderate AD	ANOVA *p*-value
Male:Female	3:12	8:9	2:9	0.24
Age	74.7 (5.2)	74.3 (7.7)	78.4 (8.6)	0.12
CDR	0	0.48 (0.2)	0.6 (0.2)	
MMSE	29.4 (1)	27.8 (1.7)	22 (1.6)	
APOE Not tested	15	3	0	
APOE ε2ε2		0	0	
APOE ε2ε3		1	0	
APOE ε3ε3		6	4	
APOE ε2ε4		0	0	
APOE ε3ε4		5	5	
APOE ε4ε4		2	2	

*Mean values are given for male to female ratios, age in years, CDR and MMSE values as well as APOE status for all participants. Standard deviations are noted in brackets and p values on the right show non-significant differences between groups. Abbreviations: AD, Alzheimer’s Disease; CDR, Clinical Dementia Rating; MMSE, Mini-Mental State Examination; APOE, Apolipoprotein E*.

### MRI Acquisition

All participants were imaged on a Siemens Tim Trio 3T scanner (Siemens Healthcare GmbH) with a 32-channel head RF array. A multimodal protocol was performed that included mcDESPOT myelin water imaging (Deoni et al., 2008), T1-weighted MP-RAGE anatomical, and diffusion tensor imaging. The mcDESPOT acquisition consists of eight variable flip angle T1-weighted spoiled gradient echo (SPGR_images and two sets of eight variable flip angle T1/T2-weighted fully-balanced steady-state free precession (bSSFP) images, with each set acquired with 0 or 180-degree RF phase cycling pattern (Deoni, 2011). In addition, an inversion-prepared (IR)-SPGR image was also acquired to correct for flip angle (B_1_ field) inhomogeneities (Deoni, 2011).

### MRI Analysis

Following the acquisition, data were visually checked for motion-related artifacts and then a standardized processing pipe-line was performed that included: (1) Skull stripping using the brain extraction tool (Smith, 2002) from the FSL software library (Smith et al., 2004); (2) Linear registration to account for subtle inter-scan motion using FSL’s linear image registration tool (Jenkinson et al., 2002; Zhang et al., 2004); (3) Calculation of the main and transmit magnetic field (B_0_ and B_1_) calibration maps (Deoni, 2011); and (4) Estimation of the VFM at each brain voxel using a stochastic region contraction approach to fit a 3-pool model to the acquired SPGR and bSSFP data (Deoni and Kolind, 2015).

All anatomical and VFM images were first loaded to the SPM12’s SUIT toolbox (Diedrichsen et al., 2009) to isolate structures of interest. For the whole brain and neocortex, we used the following steps for analysis: gray and white matter tissue class segmentation, DARTEL registration (Ashburner, 2007) to a common inter-subject space, a DARTEL utility to create Jacobian images. For the cerebellum, we used the following steps for analysis: SPM12’s SUIT toolbox (Diedrichsen et al., 2009) to isolate the structure, DARTEL registration to SUIT space, a DARTEL utility to create Jacobian images. Jacobian determinant images have previously shown promise in discriminating neurodegeneration by allowing a comparison of the expansion and contraction of voxels across and within subjects (Studholme et al., 2004a,b; Hua et al., 2009, 2010; Anderson et al., 2012; Aksman et al., 2016). After applying a thresholded mask created in Matlab to fit the whole brain, cerebrum or whole/anterior/posterior cerebellum to exclude extracerebral or extracerebellar voxels, we used a two-class linear support vector machine learning algorithm, implementing the *C* cost support vector classifier at a fixed value of *C* = 1 throughout all classifications. Within the full dataset, we defined a set that is split into a training set *N* of subjects used to train the classifier, and a set *T* of subjects that are used to test the classifier’s prediction ability. Specifically, we used leave-one-out cross-validation to learn a function from the data that can accurately predict the labels of unseen or new patterns and thus evaluate the classifier’s performance. The model parameters learned in the training phase are represented as weights (weighted images are displayed in Supplementary Figure 1) and demonstrate the relative contribution of each feature to the predictive task.

Using freely available Matlab code^1^, we created Jacobian weighted images, forward maps, as well as *p* and *t* thresholded maps at *p* ≤ 0.005. Cerebellar masks were created by using the SUIT atlas (Diedrichsen et al., 2009) by combining vermis, left and right cerebellar lobules I–V to create the anterior cerebellar mask, and vermis, left and right cerebellar lobules VI–X to create the posterior cerebellar mask. The cerebrum mask consisted of the whole brain mask but excluding the cerebellum and brainstem. Jacobian weighted GM volume will be referred to as GM volume throughout the manuscript.

## Results

**Figure 1:**
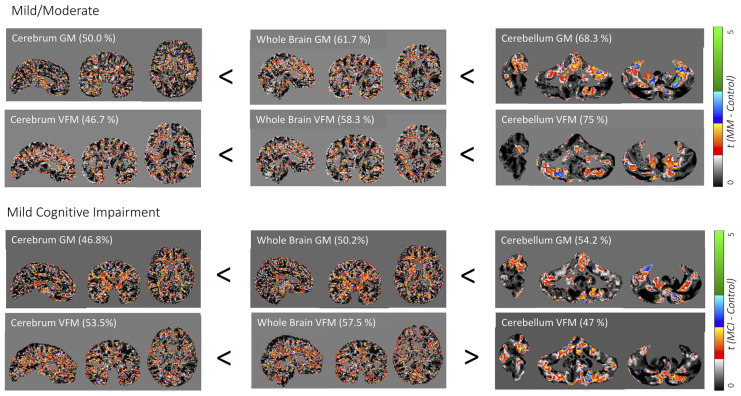
Weighted prediction *t*-maps for the whole brain and cerebellum. Jacobian gray matter (GM) volume and volume fraction myelin (VFM) prediction *t*-maps for the cerebrum, whole brain, and whole cerebellum for mild/moderate Alzheimer’s disease (AD; above) and mild cognitive impairment (MCI; below). Prediction accuracy percentage is noted in brackets and *t*-values ranging from 0 to 5 are indicated with a color bar on the right, with the lowest *t*-value in black to the highest *t*-value in green. x/y/z coordinates for the neocortex and whole-brain are 64/78/58 in DARTEL space, and 70/47/43 for the cerebellum in SUIT space.

As expected, cognitive decline increased with increasing dementia stages (Table 1). The cerebellum as a region of interest displayed up to 28% higher classification accuracy when compared with the neocortex and up to 18% higher classification accuracy when compared with the whole brain, with the highest prediction accuracy of 75% of cerebellar VFM for mild/ moderate dementia (all results thresholded at *p* ≤ 0.005, Figure 1). Clusters with the highest cerebellar effects were localized in the lateral cerebellum (x/y/z: 33/26/29 and 37/58/38 in SUIT space).

**Figure 2:**
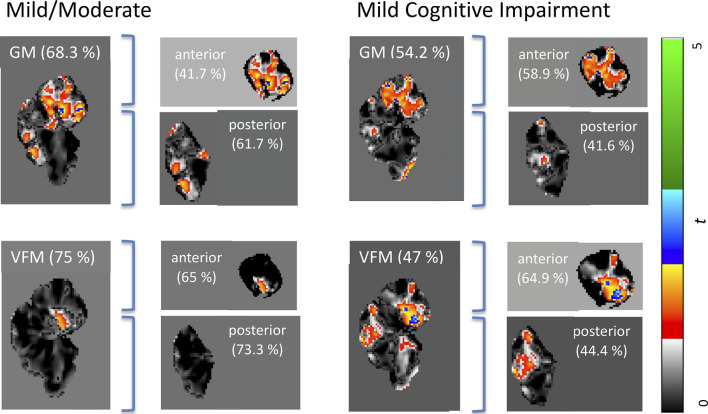
Weighted prediction *t*-maps for the whole, anterior, and posterior cerebellum. Jacobian GM volume and VFM prediction *t*-maps for the whole, anterior, and posterior cerebellum for mild/moderate AD (left), and MCI (right). Prediction accuracy percentage is noted in brackets and *t*-values ranging from 0 to 5 are indicated with a color bar on the right, with the lowest *t*-value in black to the highest *t*-value in green. All *x* coordinates are *x* = 70 in SUIT space.

Weighted images are shown in Supplementary Figure 1. While classification accuracy increased with AD stages, GM volume was the best predictor for the earliest stage of dementia (MCI), while VFM demonstrated the highest prediction accuracy for the mild/moderate stage of dementia when compared with typically aging controls. Similarly, the cerebellar GM showed the highest prediction accuracy for CDR score (66.8%; Supplementary Figure 2), and consistently higher prediction accuracy when compared with the neocortex (Supplementary Figure 2). When dividing the cerebellum into its anterior and posterior lobe, the posterior cerebellar contribution increased in both GM volume and VFM (Figure 2). These changes were strongest in the Crus I/II (Figure 2).

## Discussion

In this study, we investigated the contribution of cerebellar GM volume and VFM to predict the first two early stages of AD (MCI and mild/moderate AD) when compared with the whole brain and cerebrum gray matter volume and volume fraction myelin.

Our findings suggest VFM and GM volume loss occur in the early stages of AD, with distinct patterns of anterior and posterior cerebellar contributions (Figure 2). Specifically, disease classification was driven by differences in the posterior cerebellum with its prediction accuracy increasing with symptom severity, paralleling histopathological findings of cerebellar β in the posterior cerebellar lobe (Jacobs et al., 2018).

The cerebellum can be divided functionally into the motor and sensorimotor functions processed in the anterior- posterior lobe and cognitive functions in posterior cerebellar regions, enabled by its distinct neuroanatomic (Middleton and Strick, 2001; Kelly and Strick, 2003) and functional connectivity (Buckner et al., 2011) connections. During the development of AD, cerebellar GM volume appears to follow a predictable pattern, affecting the vermis and posterior lobe in the early stages of the disease, continuing to evolve to the anterior lobe with disease progression (Jacobs et al., 2018). Indeed, lower GM cerebellar anterior volume has been shown in patients with early-onset AD carriers compared to non-carriers (Reiman et al., 2012) as well as in patients with MCI, although the direct nature of its contribution remains unclear possibly because of the diffuse nature of the disease (for a review, Jacobs et al., 2018). In contrast, cerebellar white matter volume declines more rapidly than GM volume, a pattern similar to that observed in the cerebral hemispheres (Jernigan et al., 2001). This pattern of different white and GM volume decline was supported by our study results, where myelin water fraction demonstrated the highest prediction accuracy, underlining the role of white matter alterations at the earliest stages of dementia.

These early developments of AD are also reflected microstructurally, as more diffuse amyloid-beta deposits in early-onset patients showed cerebellar pathology 30 years earlier than sporadic patients (Cole et al., 1993). Here, the degree of cerebellar amyloid-beta was negatively correlated with the age of onset, indicating cerebellar atrophy as a possible biomarker for the early stages of AD. This cerebellar pathology in early-onset AD is further accompanied by cerebellar motor phenomena such as ataxia, especially in PSEN1 mutations (Bateman et al., 2011). Motor phenomena precede the loss of cognitive functions and are associated with early detectable cognitive impairments of MCI and incident AD (Camicioli, 2010). This is in line with our results where the anterior cerebellar contribution to AD was strongest in the early stages followed by increasing posterior cerebellar contribution with cognitive function loss as indicated by decreasing CDR values and prediction (Table 1, Figure 2, Supplementary Figure 2). Our results are further paralleled by histological findings, where the concentration of cerebellar soluble fibrillar amyloid oligomers was inversely correlated with MMSE AD classification performance but positively with the presence of cerebral plaques and tangles, suggesting that AD molecular changes possibly already occur in the cerebellum in the preclinical stages and may contribute to the symptomatology and pathophysiology of the disorder (Mann et al., 1990). While our cerebellar findings seem to mirror these histopathological findings, it remains unclear whether this pattern corresponds to microtissue changes and should be followed up by combining VFM and GM volume MRI scans and histopathology of the cerebellum on tissue from early AD stages.

Cerebellar amyloid-β oligomers have been documented to be affected in a counter-clockwise manner starting in the posterior cerebellar lobe, thus paralleling cerebrum atrophy staging and associated symptom progression (Jankowsky et al., 2004; Gentier et al., 2015), has led to an AD stage model of cerebellar atrophy starting from the posterior to the anterior lobe with disease progression (Jacobs et al., 2018). Based on our findings, we propose a refined model of cerebellar contribution to AD development. We suggest that early AD disease stages are driven by both the anterior and posterior cerebellum, but an initially higher anterior cerebellar lobe contribution to the classification of the earliest stages of the disease and posterior cerebellar lobe contribution increasing with symptom severity. This parallels cerebellar functionality and AD symptom disease progression, as motor phenomena that are classically associated with anterior cerebellar dysfunction, such as ataxia, are early AD symptoms preceding cognitive symptoms that have been associated with posterior cerebellar dysfunction.

While high prediction accuracies of up to 75% could indicate an important contribution of the cerebellum to the earliest stage of AD (mild/ moderate), it decreases for MCI to around 50%. This discrepancy might suggest a less substantial contribution of the cerebellum to MCI development. However, when investigating cerebellar sub-regions, a pattern of higher anterior cerebellar contribution to MCI classification appears (64.9% prediction accuracy; Figure 2), which could be linked to its essential role in motor function and dysfunction.

Our findings support the role of cerebellar contribution to the early stages of AD pathology and define its unique contribution to early disease development prediction as well as subregions driving this classification. However, the cerebellum is often regarded as being spared in AD pathology and is consequently usually used as a control area or reference region in PET imaging studies over total brain volume to control for differences (for example, Dukart et al., 2010). Nevertheless, when a study compared regional glucose metabolism normalization methods using either total cerebral or cerebellar volume, cerebellar normalization was determined superior in identifying dementia patients in comparison to control subjects (Dukart et al., 2010). Together with our findings, it can be suggested to rather chose global brain measures than cerebellar volume for normalization methods to avoid bias towards early AD stages.

## Data Availability Statement

The datasets generated for this study are available on request to the corresponding author.

## Ethics Statement

This study was reviewed by the IRB Department of Bradley Hospital, Rhode Island, USA. The patients/participants provided their written informed consent to participate in this study.

## Author Contributions

MB has been the lead in conceptualizing and writing the manuscript, as well as performing the analyses involved. SC, PM and SS have collected the data, recruited participants and performed the clinical interviews, as well as been involved in the design of the overall study. SD has been instrumental in feedback on the manuscript as well as adding insightful comments to the discussion and introduction, and was key to the design of the overall study. All authors contributed to the article and approved the submitted version.

## Conflict of Interest

While SD receives salary and grant support from Nestlé S.A., it does not overlap with this material.

The remaining authors declare that the research was conducted in the absence of any commercial or financial relationships that could be construed as a potential conflict of interest.

## Footnotes

^1^https://github.com/leonaksman/lpr

## Supplementary Material

SUPPLEMENTARY FIGURE 1 Weighted maps for the whole brain and cerebellum. Jacobian gray matter (GM) volume and volume fraction myelin (VFM) weighted maps for the cerebrum, whole brain, and whole cerebellum for mild/moderate AD (above) and mild cognitive impairment (below). Prediction accuracy percentage is noted in brackets and *t*-values ranging from 0 to 5 are indicated with a color bar on the right, with the lowest *t*-value in black to the highest *t*-value in green. x/y/z coordinates for the neocortex and whole-brain are 64/78/58 in DARTEL space, and 70/47/43 for the cerebellum in SUIT space.

SUPPLEMENTARY FIGURE 2 Weighted prediction *t*-maps for the whole brain and cerebellum. Jacobian gray matter (GM) volume and volume fraction myelin (VFM) weighted maps for the cerebrum, whole brain, and whole cerebellum for clinical dementia rating (CDR) scores. Prediction accuracy percentage is noted in brackets and *t*-values ranging from 0 to 5 are indicated with a color bar on the right, with the lowest *t*-value in black to the highest *t*-value in green. x/y/z coordinates for the neocortex and whole-brain are 64/78/58 in DARTEL space, and 70/47/43 for the cerebellum in SUIT space.
