# Supplementary figures and images for: Machine Learning Classification Identifies Cerebellar Contributions to Early and Moderate Cognitive Decline in Alzheimer’s Disease
Source: Front Aging Neurosci. 2020 Nov 3;12:524024. doi: 10.3389/fnagi.2020.524024 (PMC7669549; doi:10.3389/fnagi.2020.524024)

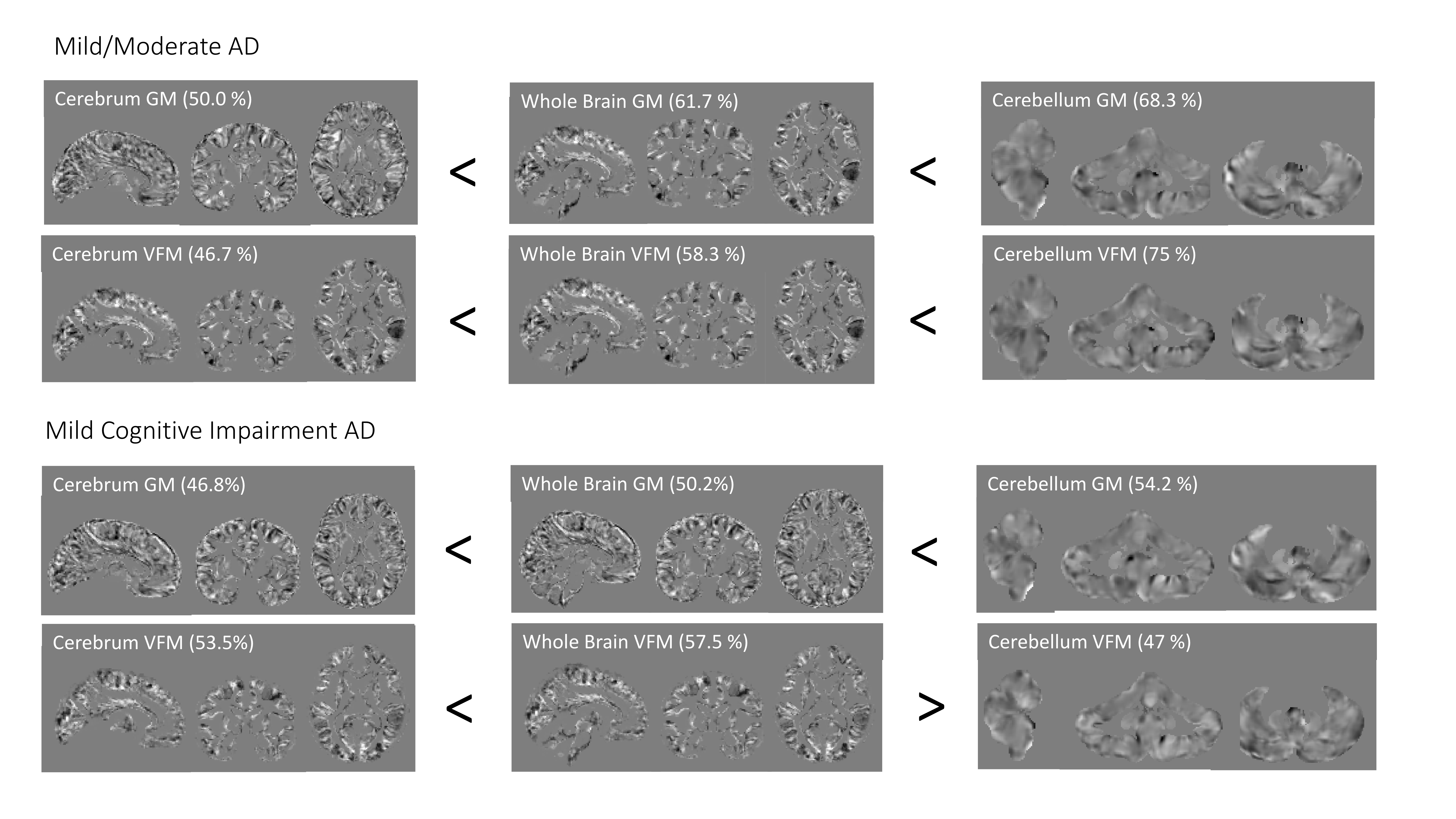

Supplement: SUPPLEMENTARY FIGURE 1 — Weighted maps for the whole brain and cerebellum. Jacobian gray matter (GM) volume and volume fraction myelin (VFM) weighted maps for the cerebrum, whole brain, and whole cerebellum for mild/moderate AD (above) and mild cognitive impairment (below). Prediction accuracy percentage is noted in brackets and t-values ranging from 0 to 5 are indicated with a color bar on the right, with the lowest t-value in black to the highest t-value in green. x/y/z coordinates for the neocortex and whole-brain are 64/78/58 in DARTEL space, and 70/47/43 for the cerebellum in SUIT space. [file Image_1.tiff]

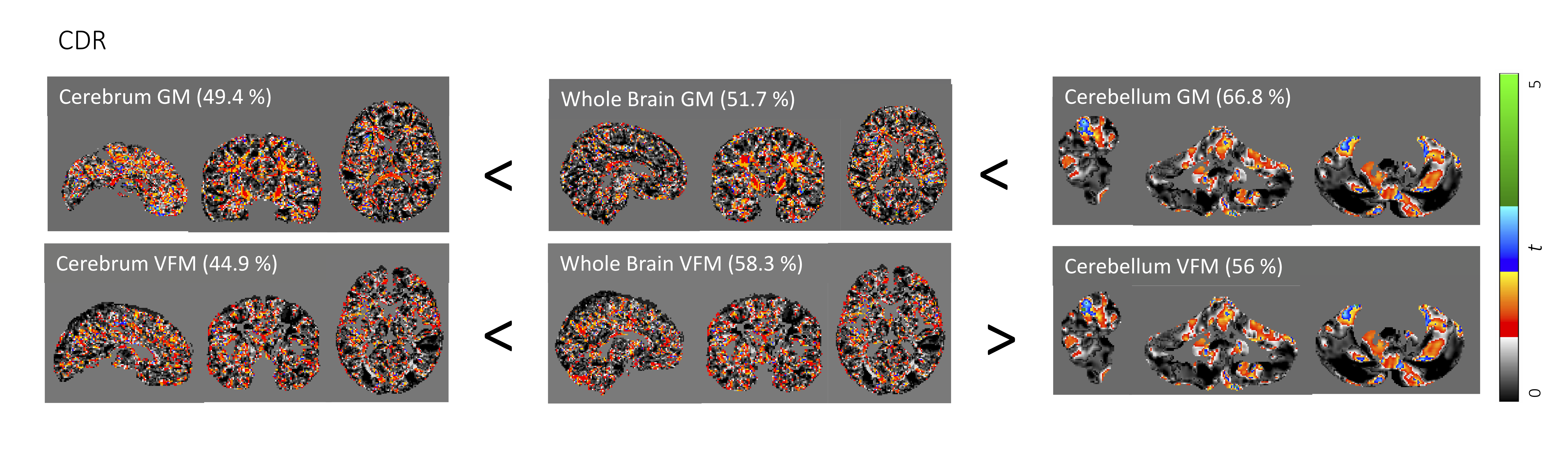

Supplement: SUPPLEMENTARY FIGURE 2 — Weighted prediction t-maps for the whole brain and cerebellum. Jacobian gray matter (GM) volume and volume fraction myelin (VFM) weighted maps for the cerebrum, whole brain, and whole cerebellum for clinical dementia rating (CDR) scores. Prediction accuracy percentage is noted in brackets and t-values ranging from 0 to 5 are indicated with a color bar on the right, with the lowest t-value in black to the highest t-value in green. x/y/z coordinates for the neocortex and whole-brain are 64/78/58 in DARTEL space, and 70/47/43 for the cerebellum in SUIT space. [file Image_2.tiff]
